# Peptide-based inhibition of CD44v6 renders liver carcinomas more susceptible to therapeutic intervention

**DOI:** 10.1007/s00109-025-02601-5

**Published:** 2025-10-31

**Authors:** Akshaya Srikanth, Ranjitha Vishnu Anand Rao, Rui Dong, Umesh Tharehalli, Thomas F. E. Barth, Klaus Dembowsky, Thomas Seufferlein, Reinhold Schirmbeck, André Lechel

**Affiliations:** 1 Department of Internal Medicine I, University Hospital Ulm, Ulm, Germany; 2 Department of Pathology, University Hospital Ulm, Ulm, Germany; 3 Amcure GmbH, Stutensee-Büchig, Germany

**Keywords:** Liver carcinoma, CD44v6, AMC303

## Abstract

**Abstract:**

Cluster of differentiation 44 variant 6 (CD44v6) has been described in various types of cancer, including liver cancer. Despite significant advancements in cancer therapy, there is still an urgent medical need for new therapeutic strategies for the treatment of liver tumours that are efficacious but well tolerated. One promising approach involves the use of small molecules, such as peptides, which intervene in central signalling pathways to prevent the migration of cancer cells and their invasion into other organs without affecting other cell types. We analysed the expression of CD44v6 in human cirrhotic livers, liver tumour tissues and organoids as well as in preclinical models of chronic liver disease and subsequent liver tumour formation. We overexpressed CD44v6 in CD44v6-negative cell lines to assess its impact on the functionality of cancer cells. Furthermore, we used AMC303, a peptide inhibitor that specifically binds to CD44v6, to investigate the consequences of CD44v6 inhibition in liver cancer and its impact on combination therapies. We demonstrated that CD44v6 is expressed in chronic liver conditions, and is overexpressed in liver cancer, where it serves as a cancer stem cell marker. We also established that CD44v6 influences migration and stemness in liver cancer, and its overexpression triggers an altered gene expression pattern including increased EMT and stemness signatures. Notably, we were able to increase the efficacy of TKI/chemotherapeutics by inhibiting CD44v6. In summary, inhibition of CD44v6 renders liver carcinomas more susceptible to therapeutic intervention, thereby representing a promising target for cotreatment strategies.

**Key messages:**

- CD44v6 is expressed in chronic liver disease and is overexpressed in liver cancer.
- CD44v6 has been demonstrated to exert a significant influence on the processes of migration and stemness in liver cancer.
- Overexpression of CD44v6 triggers an altered gene expression pattern, characterized by an increase in EMT and stemness signatures.
- Inhibition of CD44v6 increases the efficacy of TKI/chemotherapeutics in cell lines and organoids generated from liver carcinoma.
- CD44v6 inhibition renders liver carcinomas more susceptible to therapeutic intervention, thereby representing a promising target for cotreatment strategies.

**Supplementary information:**

The online version contains supplementary material available at 10.1007/s00109-025-02601-5.

## Introduction

Liver cancer represents a major health problem worldwide and is one of the most difficult types of cancer to treat. The development of new anticancer strategies is still an urgent medical need to treat this disease. The most frequently occurring liver tumours are hepatocellular carcinoma (HCC) and intrahepatic cholangiocarcinoma (iCCA).

The CD44 family of transmembrane glycoproteins comprises alternatively spliced variants that are involved in numerous cellular processes like lymphocyte maturation [1]. In the non-inflamed liver, CD44 is only expressed in cells of myeloid origin, like Kupffer cells and lymphocytes [2]. CD44 expression in hepatocytes can only be found upon malignant transformation. CD44s, the standard variant of CD44, and CD44v, representing different variant isoforms of CD44, are expressed in liver carcinoma. CD44v expression correlates with histological grading and is associated with a decreased overall survival and thus marks a worse prognosis [3]. Complete knockout of CD44 in a diethylnitrosamine (DEN)-induced mouse model of liver carcinoma shows that CD44 promotes HCC development by protecting DNA-damaged hepatocytes. It was demonstrated that CD44 expression enhanced growth factor signalling, thereby terminating p53 response, cell-cycle exit and apoptosis induced by DNA damage [4]. CD44 has further been identified as a prognostic marker for HCC [5]. The isoform CD44v6 has been established as a stem cell marker in several cancers, including colorectal cancer, pancreatic cancer and breast cancer [6–10]. Moreover, the CD44v6 isoform acts as a coreceptor for the receptor tyrosine kinases (RTKs) c-MET, RON and VEGFR-2 and has been shown to play an important role in tumour development, migration, invasion, metastasis and recurrence [11]. Altered CD44v6 expression has been shown in a number of epithelial cancers, such as head and neck squamous cell carcinoma (HNSCC), lung cancer, breast carcinoma, ovarian cancer, prostate cancer, oral cancer, laryngeal carcinoma, oesophageal squamous cell carcinoma, gastric cancer, pancreatic cancer, colorectal cancer and liver cancer [12–21]. CD44v6 could be targeted in some of these cancers using the small peptide inhibitor AMC303, which offers an effective and efficient therapeutic option [22–24]. AMC303 is a highly selective inhibitor of the splice variant CD44v6 with no effects on the other splice variants or the standard isoform CD44s [25]. It presumably binds to the shaft region of CD44v6 and thus prevents the binding and activation of RTKs. Additionally, AMC303 competes for an essential component of the variant region of CD44v6 and thereby interferes with its homoclustering and effectively blocks the interaction between CD44v6 and MET proto-oncogene (MET) [26]. AMC303 is a small cyclic peptide and has the typical pharmacokinetic properties of small peptides, e.g. short plasma half-life and renal excretion. Due to the high affinity to CD44v6 (single digit nanomolar affinity), it has a long residence time of up to 2–3 days in the tumour tissue [25]. Treatment of colorectal and pancreatic cancer cells with AMC303 resulted in an altered gene expression of the PI3K/AKT, MAPK/ERK, MET and VEGF pathways [23, 27].

A characteristic feature of cancer stem cells is an increased resistance to chemotherapy [28]. CD44 has been associated with increased resistance to standard cancer therapy in different cancers [29]. The functional inhibition of CD44 at the gene or protein level has been proven to reverse malignant behaviour and sensitize cells to chemo- and radiotherapy [30, 31]. CD44 positivity is linked to resistance to the tyrosine kinase inhibitor (TKI) sorafenib [32]. Knockdown of CD44 in HLE cells, a cell line generated from hepatocellular carcinoma, sensitized them to sorafenib in a TGFβ-dependent manner [32]. CD44 also accounts for increased resistance to doxorubicin in breast cancer and sunitinib in clear cell renal cell carcinoma [33, 34]. In breast cancer, a subset of CD44 overexpressing cells was linked to reduced sensitivity to chemotherapy, radiotherapy and endocrine therapy [35–37]. Overexpression of CD44 was associated with increased resistance of ovarian cancer cells to paclitaxel [38]. Splice variants of CD44 have also been associated with reduced sensitivity to standard therapeutic agents. Specifically, knockdown of the splice variant CD44v6 in prostate cancer cell lines enhanced chemo-/radiosensitivity by downregulation of the PI3K/AKT/mTOR and Wnt/*β*-catenin signalling pathways [17]. CD44v6-positive (CD44v6^+^) circulating tumour cells have been shown to be associated with prediction of first-line treatment failure in patients with metastatic colorectal cancer [39] suggesting an association of CD44v6 expression with chemoresistance. Several studies on colorectal cancer cell lines showed the correlation between CD44v6 expression and increased resistance to 5-FU, oxaliplatin and leucovorin, an effect that was completely reversed upon knockdown of CD44v6 [40, 41].

The precise role of CD44v6 in liver cancer and the use of peptide-based inhibition of CD44v6 are not yet analysed. In this study, we examine the expression of CD44v6 in liver cancer and its effect on certain biological cell functions. We establish that CD44v6 plays a major role in regulating EMT, migration and stemness in liver cancer cells. We also demonstrate that the specific inhibition of CD44v6 using the peptide AMC303 renders liver carcinomas more susceptible to therapeutic intervention such as TKIs and chemotherapy, therefore providing a novel therapeutic strategy to target tumours expressing CD44v6.

## Results

### CD44v6 is overexpressed in human liver carcinoma

**Fig. 1:**
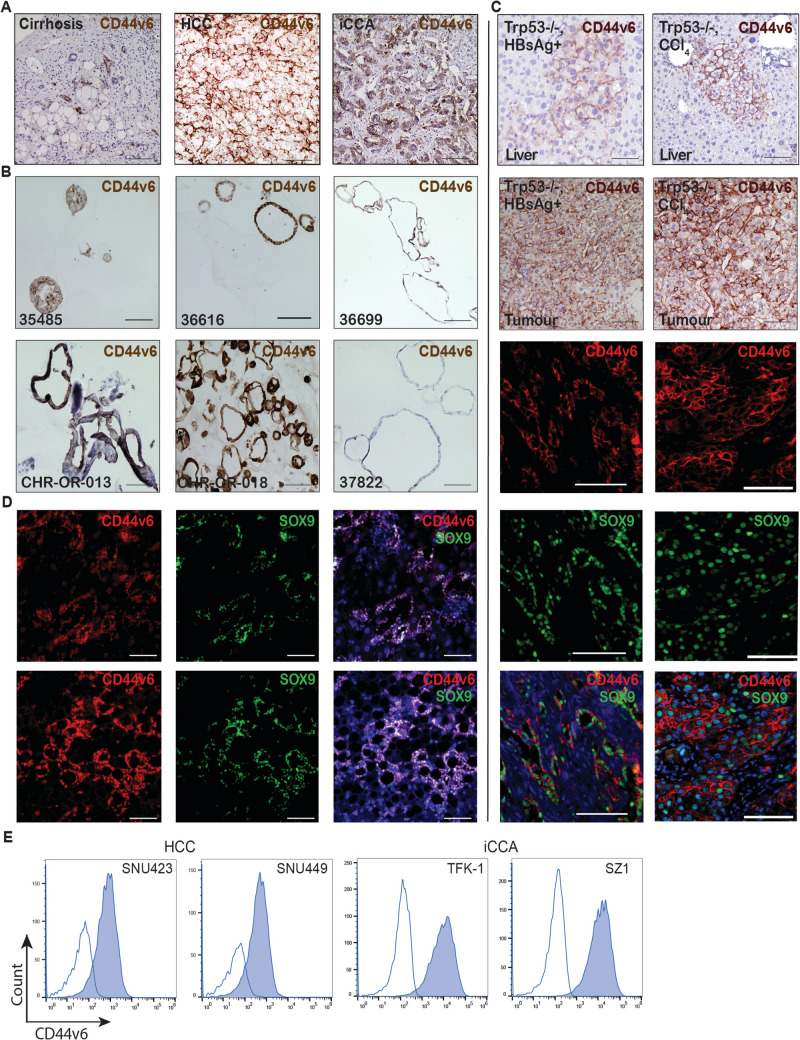
CD44v6 is overexpressed in human liver carcinoma. **A** CD44v6 expression detected by immunohistochemistry in human cirrhosis (left panel), HCC (middle panel) and iCCA (right panel). **B** CD44v6 expression in CD44v6^+^ and CD44v6^−^ (bottom right) cholangiocarcinoma organoids. A total of 5/7 cholangiocarcinoma organoids express CD44v6. **C** CD44v6 is detected in the premalignant livers and overexpressed in liver tumours from two different mouse models as shown by IHC (top and second panel). The expression of CD44v6 (red) also coincides with SOX9 (green) expression in these tumours as shown by immunofluorescence. **D** SOX9 and CD44v6 co-expression detected by immunofluorescence on human liver carcinoma sections. **E** CD44v6 expression (dark-blue histogram) in two HCC cell lines (SNU423 and SNU449) and two iCCA cell lines (TFK-1 and SZ1) detected by flow cytometry compared to the isotype control (white histogram). Scale bar: 100 µM

CD44v6 is a splice variant of CD44, whose overexpression in many cancers, including pancreatic cancer, colon cancer and breast cancer, has been shown to confer an increased migratory and invasive capacity (Table S1). In our analyses, we observed expression of CD44v6 in human cirrhotic livers, as well as in human HCC and iCCA tumours (Fig. 1A). Moreover, CD44v6 expression was also detected in 71.4% (5/7) of patient-derived cholangiocarcinoma organoids (Fig. 1B). In mouse models of chronic liver injury and subsequent tumorigenesis, we detected CD44v6 expression in the premalignant liver and in liver tumours. We showed that CD44v6 expression correlated with the expression of sex-determining region Y-box 9 (SOX9), a cancer stem cell marker in murine liver tumours (Fig. 1C). Moreover, examination of CD44v6 and SOX9 expression by IF on human liver carcinoma sections revealed a high density of CD44v6^+^ and SOX9^+^ cells (Fig. 1D). This implies that CD44v6 is enriched in human and murine models of liver cancer and may contribute to cancer stemness.

### Inhibition of CD44v6 with AMC303 impacts migration and stemness of liver tumour cells

**Fig. 2:**
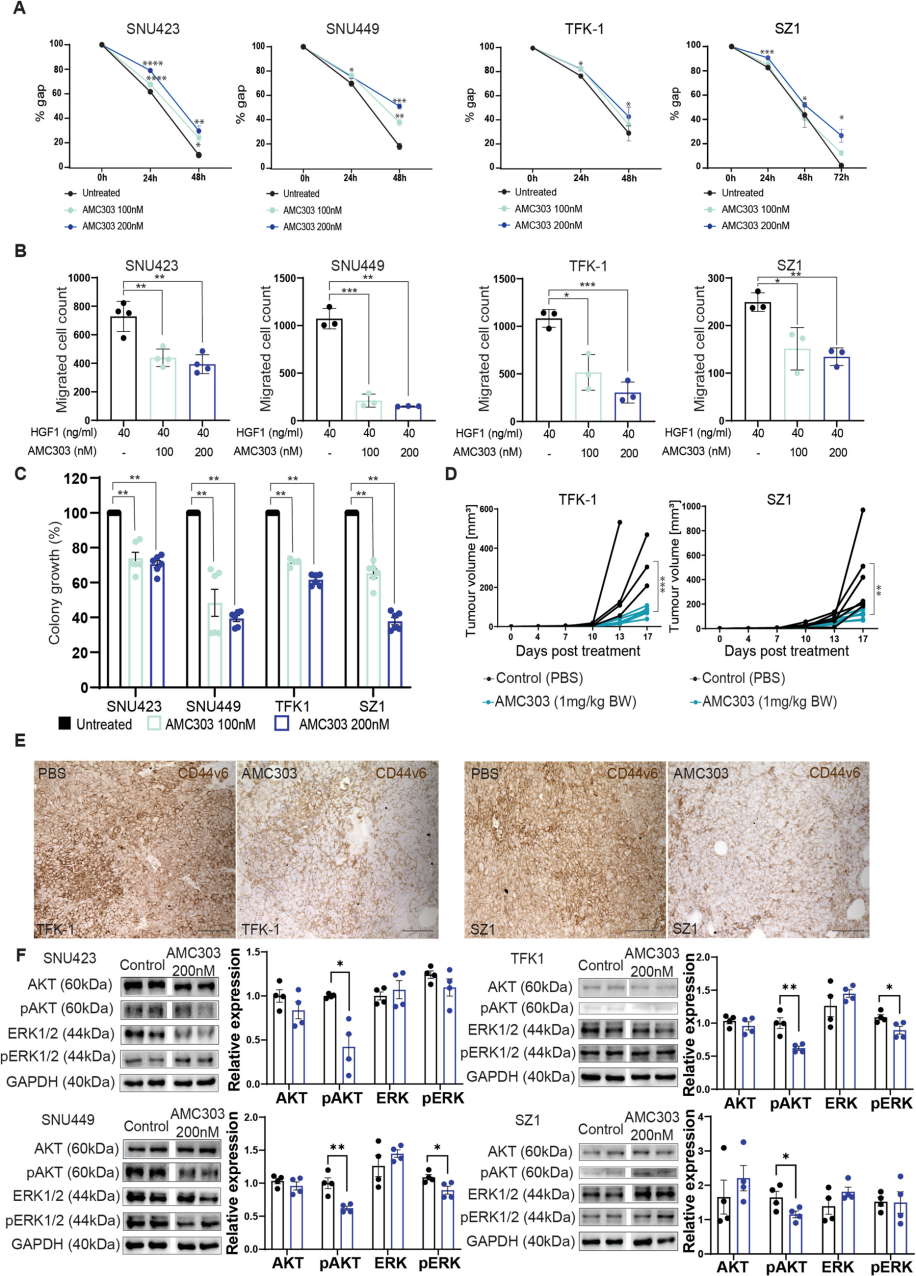
Inhibition of CD44v6 reduces migratory capacity and stemness properties of liver cancer cell lines. **A** Scratch wound healing assay showed a significant reduction in gap closure in SNU423, SNU449, TFK-1 and SZ1 upon treatment with AMC303. **B** Transwell migration assay showed a significant dose-dependent reduction in the migratory capacity towards HGF of all four cell lines upon treatment with AMC303. **C** Colony formation assay showed the formation of a significantly lesser number of colonies in all four cell lines after AMC303 treatment. **D** Reduction in tumour volume in AMC303 treated mice vs. control (PBS-treated) mice. **E** CD44v6 expression was reduced in AMC303 treated tumours compared to untreated tumours. **F** Western blot analysis of all four cell lines upon AMC303 treatment for the detection of (p)AKT and (p)ERK, with graphical representation. *n* > 3 for all experiments. *p ≤ 0.05, **p ≤ 0.01, ***p ≤ 0.001 and ****p ≤ 0.0001. Scale bar: 100 µM

Several human liver cancer cell lines were screened for the expression of CD44v6. Out of these, two hepatocellular carcinoma cell lines, SNU423 and SNU449, and two cholangiocarcinoma cell lines, TFK-1 and SZ1, were identified to express high levels of CD44v6, with > 70% of cells expressing CD44v6 on their surface (Fig. 1E). The small cyclic peptide, AMC303, directly binds to CD44v6 and thereby inhibits CD44v6 signalling [25, 26]. To test the impact of AMC303 on the migratory capacity of tumour cells, we performed a scratch wound healing assay. The percentage of gap closure was reduced in a dose-dependent manner in the CD44v6^+^ cell lines (SNU423, SNU449, TFK-1 and SZ1) treated with AMC303 (Fig. 2A). AMC303 significantly suppressed the migratory capacity of the cells. The scratch wound healing assay was also performed on the CD44v6-deficient HCC cell line, Huh7, to check the specificity of the inhibitor. AMC303 did not have any effect on the migratory capacity in Huh7 cells, confirming that it was indeed specific to CD44v6 (Fig. S1).

Furthermore, a transwell migration assay was performed to evaluate the effect of AMC303 on chemotaxis. Cells were stimulated to migrate with hepatocyte growth factor (HGF). We measured endogenous HGF levels in the conditioned medium of the cancer cells by ELISA at 24 h, 48 h and 72 h after plating the cells, to exclude an effect of endogenous HGF secretion. We observed 62–94 pg/ml in SNU423, 61–78 pg/ml in SNU449, 68–102 pg/ml in TFK-1 and 72–95 pg/ml in SZ1 cells (Fig. S2). Cancer cells were treated with 40 ng/ml of HGF, which was manyfold higher than the HGF concentration in the conditioned medium. Treatment of the cells with AMC303 significantly inhibited the migratory effect observed upon subjecting the cells to HGF in a dose-dependent fashion (Fig. 2B). This data further corroborated the biological effect of AMC303 on the migratory capacity of CD44v6^+^ cells. A colony formation assay was performed to measure the stem-cell-like potential within each cell line. A significant reduction in the colony-forming potential of the cells was observed upon treatment with AMC303 compared to untreated cells in all four liver carcinoma cell lines (Fig. 2C). This demonstrated that the stem cell composition of AMC303-treated cells is lesser compared to untreated cells, indicating that AMC303 impacts the stemness characteristics of CD44v6^+^ liver cancer cell lines.

To study the tumour-initiating potential of CD44v6^+^ liver cancer cells in vivo, 1 × 10^6^ liver carcinoma cells were subcutaneously injected into the flanks of immunocompromised NSG mice. The mice were then treated thrice a week with AMC303 by intravenous injection. We observed a significantly reduced outgrowth of tumour cells in the AMC303-treated mice as compared to control mice (injected with PBS, Fig. 2D). This reduction in the volume of tumours developed by the AMC303-treated mice indicated a reduction in the stem cell component of the cell lines. Mice treated with AMC303 expressed lower amounts of CD44v6 in the subcutaneous tumours compared to the mice treated with PBS (Fig. 2E).

Since CD44v6 acts via the PI3K and MAPK pathways, the expression levels of AKT and ERK were investigated through Western blot (Fig. 2F, Figs. S3, S4, S5, S6). While the expression of total AKT and total ERK remained the same in the AMC303-treated and -untreated cells, there was a significant reduction of phosphorylated AKT (p-AKT) expression in the treated cells. This indicated that CD44v6 is linked to the PI3K–AKT pathway, and the inhibition of CD44v6 causes a downregulation of AKT in liver carcinoma cell lines.

### AMC303 treatment rescues increased tumorigenic effects caused by CD44v6 overexpression

**Fig. 3:**
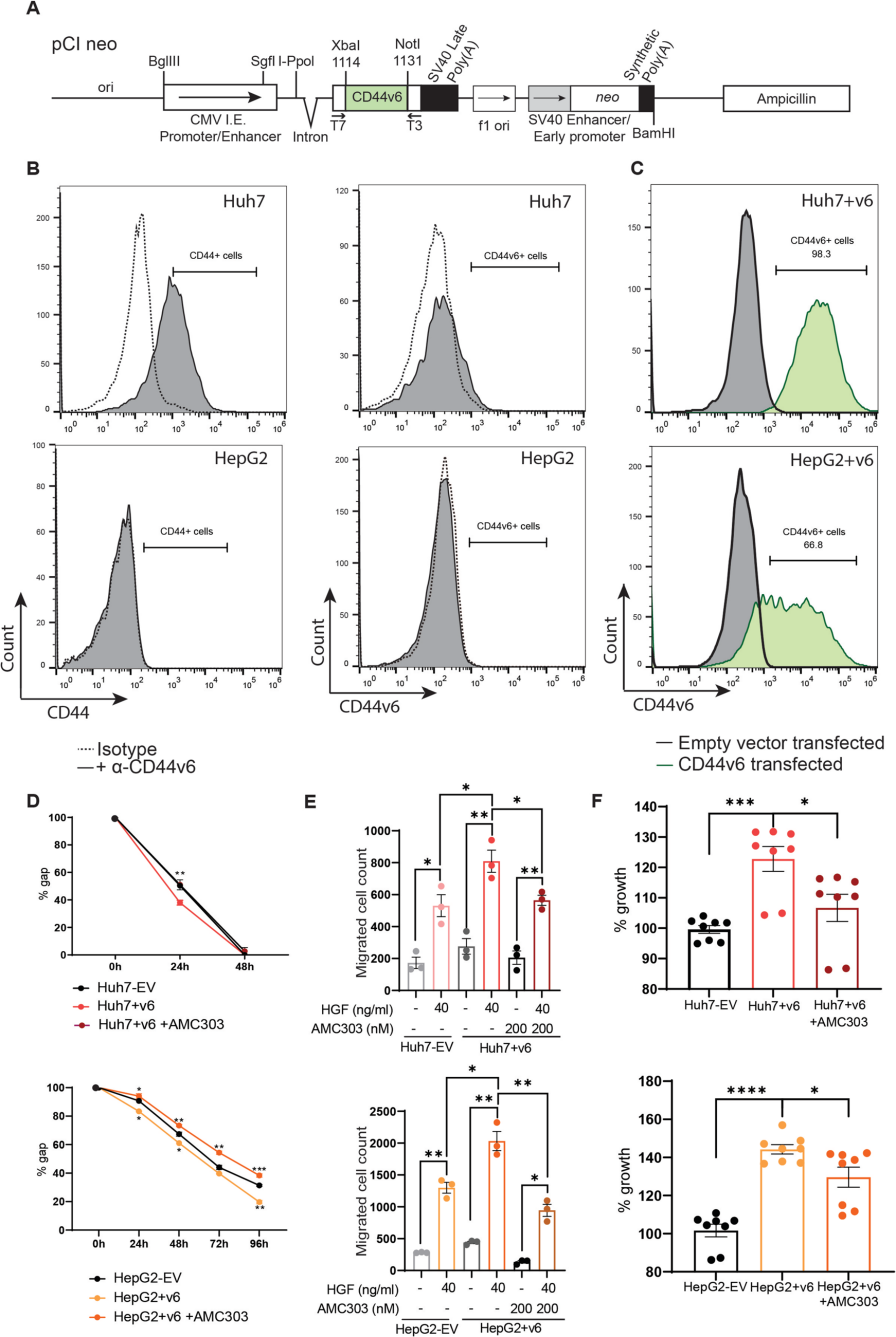
CD44v6 overexpression increased tumour-associated properties, rescued by treatment with AMC303. **A** Vector map denoting the cloning of CD44v6 into the pCIneo expression vector. **B** CD44 and CD44v6 expression in two cell lines, Huh7 and HepG2, by FACS showed that both cell lines were negative for CD44v6 expression. Dotted lines represent isotype control; solid lines represent the addition of anti-CD44v6 antibody. **C** CD44v6 expression shown by FACS was induced after transfection of cell lines with pCI-neo (black) and pCI-neo + CD44v6 (red) and sorting. **D** Scratch wound healing assay showed higher migratory potential of Huh7 + v6 and HepG2 + v6 compared to respective empty vector (EV) controls. This effect was lost when the CD44v6 overexpressing cells were treated with 200-nM AMC303. **E** Transwell migration assay showed higher migratory potential of Huh7 + v6 and HepG2 + v6 compared to EV controls in the presence or absence of HGF. A complete rescue of the increased migration was observed upon treatment of the Huh7 + v6 and HepG2 + v6 cells with AMC303. **F** Colony formation assay showed significantly higher growth rates in Huh7 + v6 and HepG2 + v6, and a reversal of this effect was seen upon treatment of these cells with AMC303. *n* = 3 for all experiments. *p ≤ 0.05, **p ≤ 0.01, ***p ≤ 0.001 and ****p ≤ 0.0001

To unequivocally show the biological effects of CD44v6 expression, CD44v6 was overexpressed in two CD44v6-negative (CD44v6^−^) HCC cell lines, Huh7 and HepG2, by stable transfection with a pCI neo-CD44v6 vector expressing the human CD44v6 gene under a hCMV promoter (Fig. 3A). The stable neomycin-resistant transfectants, Huh7 + v6 and HepG2 + v6, were compared to Huh7-EV (transfected with empty vector) and HepG2-EV, respectively. CD44v6 was stably expressed on the surface of Huh7 + v6 and HepG2 + v6 cell lines, as confirmed by FACS (Fig. 3B and C). Furthermore, the Huh7 + v6 and HepG2 + v6 cells were treated with AMC303 to observe the effect of inhibition of CD44v6 in these cell lines. A scratch wound healing assay revealed that in both cell lines, overexpression of CD44v6 led to an increase in the percentage of gap covered at every measured time point (Fig. 3D*)* compared to the EV controls. Moreover, a significant reduction in the migratory capacity, equivalent to the EV controls, was observed upon treatment of these cells with AMC303. A transwell migration assay showed a significant increase in the number of migrated cells towards HGF in Huh7 + v6 and HepG2 + v6 compared to the empty vector transfectants (Fig. 3E). Most interestingly, a significant difference was also observed in the migratory capacity of Huh7 + v6 and HepG2 + v6 cells compared to Huh7-EV and HepG2-EV cells, indicating a more potent impact of CD44v6 expression on the migration potential of liver carcinoma cell lines. Once again, a significant rescue effect of CD44v6 inhibition by AMC303 treatment was observed in both cell lines. Overexpression of CD44v6 also resulted in a highly significant increase in the number of colonies formed in a colony-formation assay, which may indicate a stem cell population within a cell line (Fig. 3F). Indeed, this characteristic was recovered upon treating the Huh7 + v6 and HepG2 + v6 cells with AMC303.

### CD44v6 overexpression is directly linked to an increased EMT and stemness signature

**Fig. 4:**
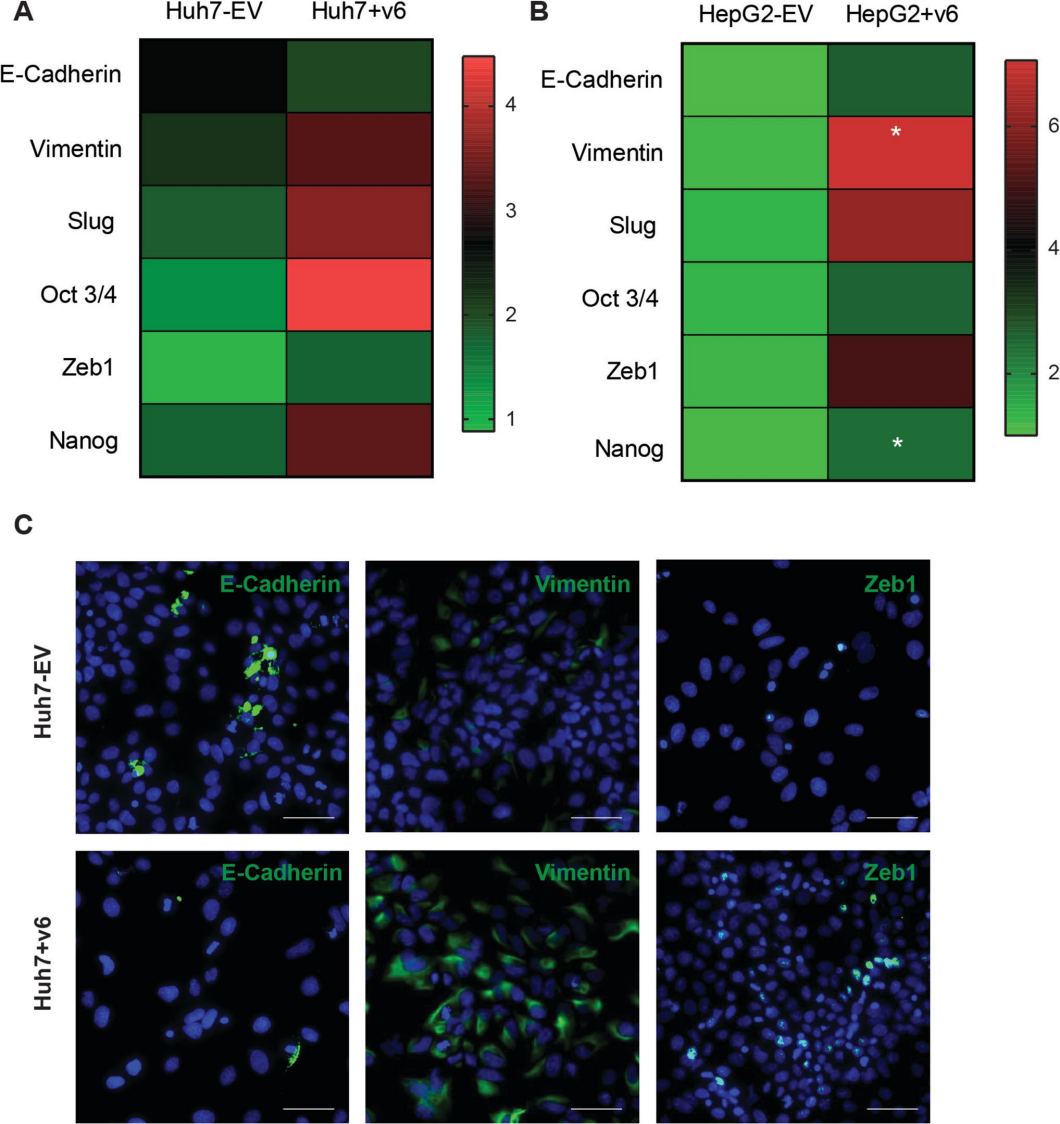
CD44v6 overexpression increased EMT and stemness signature. A general increase in EMT and stemness was observed via qPCR in the Huh7 + v6 (**A**) and HepG2 + v6 (**B**) cells compared to EV controls. The numbers on the colour scale represent the fold change relative to the housekeeping gene (RNA polymerase II). **C** Immunofluorescence detection of E-cadherin, vimentin and Zeb1 in Huh7-EV (top panel) vs. Huh7 + v6 (bottom panel), *n* = 3 for all experiments. *p ≤ 0.05. Scale bar: 100 µM

As demonstrated in a DEN-induced liver carcinoma model, CD44 surface expression and HCC initiation were mechanistically linked with the DNA-damage-induced p53 response, associated with cell-cycle exit and apoptosis by an enhanced growth factor signalling [4]. Therefore, we determined the gene expression profile of stable CD44v6-transfected cell lines, Huh7 + v6 and HepG2 + v6 versus CD44v6^−^ Huh7 and HepG2 controls by qRT-PCR. Most interestingly, the mesenchymal markers *vimentin*, *ZEB1* and *Slug* as well as the stemness markers *OCT3/4* and *NANOG* were upregulated in the CD44v6-transfected cells compared to the EV cells (Fig. 4A and B). Additionally, we confirmed an upregulation in the EMT profile of CD44v6-transfected cells by immunofluorescence (Fig. 4C). E-Cadherin expression was downregulated, while vimentin and Zeb1 expressions were upregulated in the transfected cells compared to the controls. This suggested a direct link between CD44v6 expression and the overall gene expression profile, migratory capacity, tumorigenicity, and stemness of CD44v6^+^ tumour cells.

### CD44v6 inhibition in liver cancer confers increased susceptibility to therapeutic intervention

**Fig. 5:**
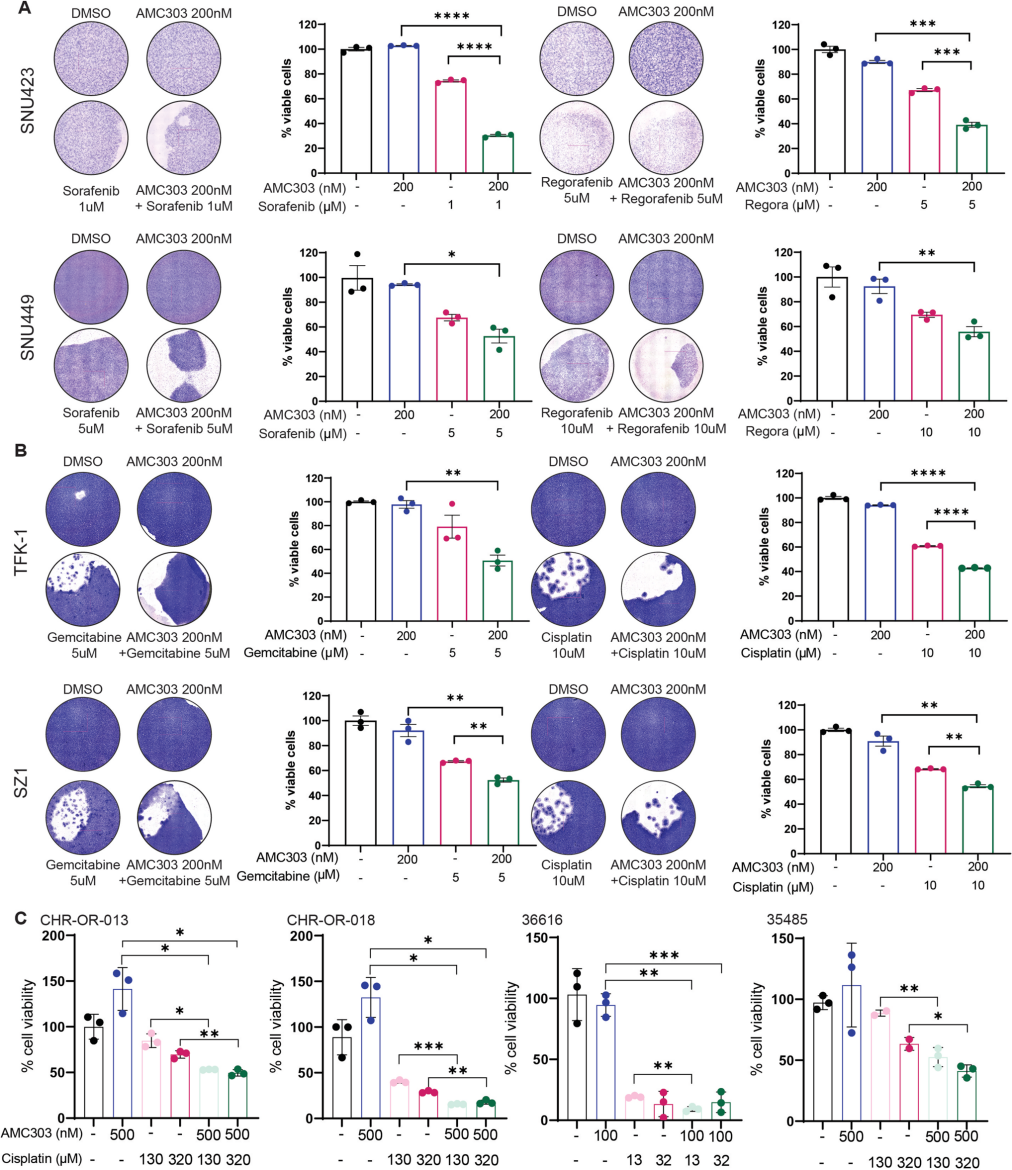
Cotreatment of AMC303 with TKI/chemotherapeutic agents increases its efficacy. Cells/organoids were pretreated with 200-nM AMC303 for 3 days, followed by treatment with the indicated drug(s). **A** The HCC cell lines (SNU423 and SNU449) were treated with 200-nM AMC303, the TKIs sorafenib (1 µM for SNU423 and 5 µM for SNU449), regorafenib (5 µM for SNU423 and 10 µM for SNU449) or a combination of AMC303 and either of the TKIs. **B** The iCCA cell lines (TFK-1 and SZ1) were treated with gemcitabine (5 µM) and cisplatin (10 µM) for 4 days, individually or in combination with 200 nM of AMC303. Media were replaced every day with a fresh supplementation of the respective drugs. On day 7, cells were fixed and stained with Giemsa, and images were taken of the stained cells. A clear reduction in the viable cell population was observed in the wells subjected to the cotreatment as compared to the single treatment wells in all four cell lines, irrespective of the TKI or chemotherapeutic agent used. **C** Cholangiocarcinoma organoids expressing CD44v6 were subjected to cotreatment with AMC303 and gemcitabine, and the cell viability was measured. The combination of the two drugs significantly improved the efficacy of treatment compared to the single treatments. *n* = 3 for all experiments. *p ≤ 0.05, **p ≤ 0.01, ***p ≤ 0.001 and ****p ≤ 0.0001

Considering that CD44v6 expression enhanced EMT and stemness, we hypothesized that exposure to AMC303 might reduce the stem cell population within the analysed cell lines, thereby making the cells more susceptible to alternate forms of treatment. To this end, CD44v6^+^ liver carcinoma cell lines were subjected to a pretreatment with AMC303 followed by treatment with standard-of-care therapeutic agents. The HCC cell lines SNU423 and SNU449 were treated with AMC303 in combination with TKIs sorafenib and regorafenib. Cells were pretreated with AMC303 for 3 days prior to the application of the TKIs. Cotreatment of the HCC cell lines with 200-nM AMC303 and 1-µM (SNU423) or 5-µM (SNU449) sorafenib as well as 5-µM (SNU423) or 10-µM (SNU449) regorafenib led to a significant reduction in cell viability (Fig. 5A), indicating that CD44v6 inhibition sensitizes HCC cells to VEGFR, PDGFR and RAF kinase inhibitors. Similarly, the cholangiocarcinoma cell lines TFK-1 and SZ1 were treated with AMC303 in combination with chemotherapeutic agents (cisplatin and gemcitabine), since they are currently used as a standard of care in the treatment of CCA. Cotreatment of both CCA cell lines with 200-nM AMC303 and 5-µM gemcitabine or 10-µM cisplatin significantly increased efficacy compared to single treatment (Fig. 5B). Human cholangiocarcinoma organoids expressing CD44v6 (see Fig. 1B) were also tested for measuring the efficacy of cotreatment with AMC303. The cell viability of the organoids treated with both AMC303 and gemcitabine was significantly reduced compared to the single drug treatments (Fig. 5C). This demonstrated that AMC303 sensitizes cholangiocarcinoma cells and patient-derived organoids to chemotherapy.

Taken together, we were able to demonstrate that inhibition of CD44v6 using AMC303 rendered liver carcinoma cells and organoids susceptible to chemotherapeutic agents and TKIs.

## Discussion

In the current study, we have characterized the expression of CD44v6 in liver tumours. We demonstrated the overexpression of CD44v6 in CD44v6^−^ liver cancer cell lines, followed by a specific peptide-based inhibition of CD44v6. We analysed the molecular biological changes due to the inhibition of CD44v6, which sensitized the tumour cells to further treatment and elucidated the effects of combination therapies in the context of CD44v6 targeting.

CD44 is a surface molecule whose expression is described on several tumour entities and is associated with reduced survival. The expression of CD44 and its isoforms has also been associated with a poor prognosis in liver carcinomas [3]. In a murine model, whole body CD44 knockout resulted in reduced induction of liver carcinomas following DEN-induced carcinogenic liver injury [4]. In particular, the CD44v6 isoform has often been described as associated with reduced survival in various tumour entities and has been specifically targeted. Among primary liver cancers, 27–30% of the cancers tested positive for the expression of CD44v6. Moreover, the expression of CD44v6 was not observed in adjacent liver tissue in any of the patients, indicating that CD44v6 expression was indeed related to malignant transformation [3, 42]. Correspondingly, we observed the expression of CD44v6 in cirrhotic human livers, and its overexpression in murine and human liver tumours, and patient-derived liver tumour organoids. Notably, we observed CD44v6 expression in the same cells that express the progenitor/stem cell marker SOX9 in both human and murine tumours (Fig. 1D), supporting the idea that CD44v6⁺ cells represent a progenitor-like subpopulation in liver tumorigenesis.

### CD44v6 in the context of EMT and stemness

CD44v6 has been reported to be responsible for the maintenance of stemness characteristics in various cancers such as colorectal cancer, breast cancer and pancreatic cancer [9, 23]. Here, we demonstrated a reduced migratory and colony-forming potential of hepatocellular and cholangio-carcinoma cell lines in vitro and impaired tumour-forming ability in vivo associated with inhibition of CD44v6, along with an upregulation of cancer stem cell-related genes (OCT3/4, NANOG) upon overexpression of CD44v6. Current data suggest that CD44v6 plays a crucial role in the interaction between stem cells and their niche, which supports the notion that stem cells are kept (held back) in their niches, but the exact mechanism for stem cell modulation by CD44v6 is still unknown. Some studies have shown that the microenvironment, in conjunction with the signals generated by hepatocyte growth factor, osteopontin and stromal cell-derived factor 1, contributes to the activation of the Wnt/*β*-catenin and PI3K/AKT signalling pathways, which confer metastatic activity to tumorigenic cells and promote survival signalling in cancer stem cells [9]. The expression of CD44v6 in colorectal cancer also correlates inversely with the histological differentiation of tumours and is itself a cancer stem cell marker [43]. Our data strongly suggest that CD44v6 promotes cancer stemness in hepatobiliary carcinomas, which makes the inhibition of CD44v6, and thus the reduction of cancer stem cells, an interesting target for therapy.

### Inhibition of CD44v6 in liver cancer is a promising tool in the use of combination therapies

Due to its restriction to proliferative tissues and upregulation in a variety of cancers, CD44v6 is a prime target for cancer therapy. A number of strategies targeting CD44 and its isoforms have been conceived [44]. The use of monoclonal antibodies to target CD44v6 was tested in HNSCC. However, due to the high toxicity and low benefit of the treatment, clinical development in this direction was discontinued [45]. Over the last few years, several preclinical studies have also been conducted in which various monoclonal antibodies against CD44 and CD44v6 were investigated in pancreatic cancer, acute myeloid leukaemia, breast cancer and chronic lymphocytic leukaemia [46–48]. Peptides have been discovered with a minimum length of five amino acids containing a critical sequence that targets CD44v6 and are sufficient to inhibit vascularization of pancreatic tumours [26, 27]. The same sequence is contained in a cyclic 5-mer peptide (AMC303) that was used in our study to inhibit CD44v6 in human liver carcinoma cell lines.

Increased resistance to chemotherapy is a feature exhibited by cancer stem cells [28]. The functional inhibition of CD44 and some of its splice variants at the gene or protein level has been proven to reverse some malignant behaviour and sensitize cells to standard-of-care treatment regimens, such as chemo- and radiotherapy. The knockdown of CD44v6 in prostate cancer cell lines has been demonstrated to enhance chemo/radiosensitivity, with the underlying mechanism involving the downregulation of the PI3K/AKT/mTOR and Wnt/*β*-catenin signalling pathways [17]. Several studies on colorectal cancer cell lines have demonstrated a correlation between CD44v6 expression and increased resistance to 5-FU, oxaliplatin, and leucovorin. This effect was found to be completely reversed upon the knockdown of CD44v6 [40, 41]. In the current study, we demonstrate that the inhibition of CD44v6 increased the susceptibility of liver cancer cells and organoids to the chemotherapeutics cisplatin and gemcitabine and the receptor tyrosine kinase inhibitors, sorafenib and regorafenib. Mortensen et al. showed that targeting CD44v6 using a radioactive iodine-labelled antibody (^131^I-AbN44v6), in combination with sorafenib, greatly reduced the spheroid growth of thyroid carcinoma in vitro [49, 50]. The use of the lead compound silibinin, targeting CD44v6, along with 5-FU to treat the colon carcinoma cell line HCT116, led to a synergistic effect that suppressed migration and stemness, activated apoptosis and inhibited PI3K/MAPK signalling to a higher degree than standalone treatment [51]. Therefore, the combination of CD44v6 inhibition with other anticancer agents holds the prospect of a potent therapeutic strategy.

One of the strategies for addressing the resistance to standard cancer therapies exhibited by cancer stem cells, such as CD44v6^+^ cells, is to induce the differentiation of these cells. In the present study, the liver carcinoma cell lines and patient-derived organoids were pretreated with the CD44v6 inhibitor, AMC303, for a period of 72 h prior to stratification for combined therapy. This was done in order to induce differentiation of cells and activate anti-resistance markers that would render the cells more susceptible to the alternate therapies. It has been previously demonstrated that the knockout of CD44v6 in colorectal cancer cells induced the differentiation of cancer-initiating cells, which resulted in a decrease in sphere-forming ability and a downregulation of stemness genes related to cancer-initiating cells [40]. These findings complemented our results, indicating that the inhibition of CD44v6 results in the loss of EMT and stemness, which could enhance the efficacy of standard therapeutics. While our combination treatment findings were obtained in vitro and in patient-derived organoids, they provide a strong rationale for future in vivo studies to validate the efficacy of CD44v6-targeted co-therapy in a complete tumour microenvironment.

Taken together, we have successfully identified therapeutic interventions that can be employed in conjunction with CD44v6 inhibition to combat the lethal disease of liver cancer. This may represent a novel avenue for the translation of therapeutics into the treatment of liver cancer.

## Materials and methods

### Mice

Mice with liver-specific Trp53 deficiency (abbreviated as Trp53−/−) were generated as previously described [52]. Trp53−/− mice were crossed with Alb-HBs^+^ mice (abbreviated as HBsAg +), which express the hepatitis B surface antigen under the liver-specific albumin promoter [53]. In the second model, Trp53−/− mice were treated at the age of 6 weeks, intraperitoneally with 0.5 ml/kg body weight CCl_4_ (T5648-5G, Sigma-Aldrich) dissolved in olive oil (1:3) twice a week over a time period of 16 weeks. Both mouse models are models of chronic liver disease and subsequent liver carcinoma formation. Subcutaneous transplantation experiments were performed in NSG mice (NOD.Cg-Prkdc^scid^ Il2rg^tm1Wjl^/SzJ). All mice were maintained in a specific pathogen-free environment. All animal experiments were previously approved and performed in accordance with the University Animal Care Committee and the Federal Authorities for Animal Research (35/9185.81-3.81, Regierungspraesidium Tuebingen, Baden-Wuerttemberg, Germany).

### Immunohistochemistry (IHC)

Formalin-fixed paraffin-embedded sections were deparaffinized in xylene and then rehydrated in receding concentrations of isopropanol. Antigen retrieval using EDTA buffer was achieved by heating the buffer in a microwave. The sections were blocked with Dual Endogenous Enzyme Block (K4065, Dako) for 5 min, followed by SuperBlock solution (37536, Thermo Scientific) for 30 min at room temperature, and then incubated overnight with the primary antibody (MAB4073, Merck) at a 1:1430 dilution. Sections were incubated for 40 min with labelled polymer HRP (K4065, Dako) at room temperature. DAB Vector was used to visualize the positive signals. A total of 20% haematoxylin was used as a counterstain. Finally, the sections were dehydrated by passing them through a series of isopropanol of increasing concentrations and then through xylene before mounting.

### Immunofluorescence staining

Immunofluorescence staining was performed on murine and human FFPE sections to detect CD44v6 and SOX9. The tissues were deparaffinized and rehydrated. Antigen retrieval was performed using citrate buffer for murine tissues and EDTA buffer for human tissues in a microwave. The sections were blocked using 10% goat serum (S2000, Biowest). The samples were incubated overnight at 4 °C with the primary antibodies (CD44v6: BMS125, Invitrogen, 1:200; mSOX9: AB5535, Millipore, 1:500; hSOX9: Santa Cruz, sc-20095, 1:100). The samples were then incubated for 2 h at room temperature with the respective secondary antibodies (anti-rat (A10522, Thermo Fisher Scientific), 1:500; anti-rabbit (A11034, Thermo Fisher Scientific), 1:250; anti-mouse (Sigma), 1:400; and anti-rabbit (Zymed), 1:400). The slides were mounted with Antifade Gold DAPI Mounting Medium and observed under the microscope (BZ-9000, Keyence).

### Cell culture

HCC cell lines HepG2 (HB-8065) and SNU423 (CRL-2238) were purchased from ATCC, while Huh7 was obtained from Thermo Fisher Scientific. SNU449 (CRL-2234) was a gift from Frank Tacke, Hepatology and Gastroenterology, Charité Berlin, and TFK-1 (ACC 344) and SZ1 were gifts from Ruben Plentz, Internal Medicine I, University Hospital Tübingen. All cell lines except HepG2 were cultured in RPMI1640 media (21875-034, Gibco) supplemented with 10% FBS (P30-3031, PAN Biotech) and 1% penicillin–streptomycin (15140-122, Gibco). HepG2 cells were cultured in DMEM (4195-039, Gibco). The cells were maintained in a humidified 5% CO_2_ incubator at 37 °C. The cells were regularly monitored for *Mycoplasma* contamination and deemed *Mycoplasma*-free.

### Enzyme‑linked immunosorbent assay (ELISA) for HGF

TFK1, SZ1, SNU423 and SNU449 cells were incubated for 24, 48 and 72 h. Supernatant was collected, and human HGF concentrations were quantified using a sandwich ELISA (Human HGF ELISA Kit, no. RAB0212, Sigma‑Aldrich) following the manufacturer’s instructions. Standards and samples were run in duplicate. Briefly, kit standards prepared from recombinant HGF and appropriately diluted samples (1:2) were added to the pre‑coated 96‑well plate and incubated for 2.5 h at room temperature with gentle shaking. After washing, the supplied detection reagents were applied, signal was developed with TMB One-Step substrate reagent, and the reaction was stopped with stop solution. Absorbance was read at 450 nm.

### Treatment of human cell lines with AMC303

AMC303 is a small cyclic peptide inhibitor of CD44v6. It was procured with permission from amcure GmbH, Karlsruhe. Human cell lines were treated with 100 nM or 200 nM of AMC303, as specified.

### Flow cytometry

Fluorescently labelled anti-CD44v6 (566803, BD Biosciences) was diluted 1:100 in PBS and added to the cells. The cells were then incubated in the dark at 4 °C for 1 h. Afterwards, cells were centrifuged at 1500 rpm for 5 min, the supernatant discarded, and cells were washed twice with FACS buffer. The cells were finally resuspended in FACS buffer, and the expression of different proteins was measured using an Attune^™^ NxT Flow Cytometer. Results were analysed using FlowJo.

### Scratch wound healing assay

The scratch assay or wound healing assay is designed to qualitatively measure the migratory potential of cells. A total of 5 × 10^5^ cells were resuspended in 1-ml media. A total of 70 µl of the cell suspension was added to each chamber of an ibidi culture insert (no. 81176, ibidi). The cells were allowed to adhere overnight. The silicon insert was then removed using sterile forceps, creating a gap or ‘wound’. The cells were washed once with PBS and then supplemented with 2-ml media, with or without AMC303. Images were taken using a microscope (BZ-9000, Keyence) at 0 h and then every 24 h until the gap was completely closed. The width of the gap was measured using ImageJ software.

### Transwell migration assay

Cells were seeded onto 8-μm-pore Boyden chambers (353097, Falcon) at a density of 5 × 10^4^ cells/chamber. The Boyden chambers were inserted into a 24-well plate (353504, Falcon), which contained 500-µl DMEM with or without 40 ng/ml of hepatocyte growth factor (HGF, PRSI92-470, VWR Chemicals). The cells were simultaneously treated with AMC303. The plates were incubated for 24 h in a CO_2_ incubator. Following this, media was aspirated, and the cells were fixed in the Boyden chambers using 4% ice-cold PFA. The wells and inserts were washed twice with PBS, and the inserts were gently cleaned using a Q-tip. The cells were stained with DAPI and visualized under the microscope (BZ 9000, Keyence). The cells were counted using ImageJ software (v.1.52a Rasband, W.S., ImageJ, US National Institutes of Health, Bethesda, Maryland, MD, USA, https://imagej.nih.gov/ij/, 1997–2018).

### Colony-formation assay

The colony-forming ability of cells determines their stemness component. Cells were seeded at a density of 1000 cells/well in a 6-well plate and supplemented with 2-ml media. The cells were monitored over 3 weeks, with media change and inhibitor supplementation every alternate day. After 3 weeks, the cells were washed twice with ice-cold PBS and fixed with ice-cold 100% methanol. Once the methanol dried out, cells were incubated with a 20% v/v Giemsa solution in PBS for 20 min with constant shaking. The Giemsa solution was then removed, and the cells were washed with dH_2_O. The wells were allowed to dry overnight. The Giemsa stain that was absorbed by the cells was then dissolved using 10% v/v acetic acid. A colorimetric measurement of this solution was then performed in triplicates by measuring the absorbance at 590 nm using an Infinite 200 PRO plate reader (Tecan, Switzerland).

## In vivo tumour initiation assay

Human liver carcinoma cell lines, TFK-1 and SZ1, were counted and resuspended in 1× DPBS at a density of 1 × 10^6^ cells/100 µl. The prepared cells were subcutaneously injected into the flanks of NSG mice. The following day, the mice were intravenously injected through the tail vein with 100 µl of PBS or 1 mg/kg body weight of AMC303. The mice were injected three times a week until they were analysed. The tumour volumes were measured with Vernier callipers every 3 days, until the diameter of the tumours reached 1 cm. The mice were then dissected, and the tumours harvested for further analysis.

### Molecular cloning and transfection

The human CD44v6 protein sequence was obtained from NCBI. An optimized codon sequence was created with the GeneArt software (Thermo Fisher) and cloned into the pCI neo expression vector. The vector was then linearized and transfected into Huh7 and HepG2 cells by Lipofectamine transfection. The transfection efficiency (30–40%) was measured by FACS using an anti-CD44v6 antibody (no. 566803, BD Biosciences). The transfected cells were selected using neomycin and then sorted at the Core Facility Cytometry, Ulm University, to generate a homogenous population of CD44v6-expressing cells (Huh7 + v6 and HepG2 + v6). CD44v6 was stably expressed in Huh7 + v6 and HepG2 + v6 cell lines, which were confirmed by FACS.

### qRT-PCR

RNA was isolated from cells using the Qiagen Mini Kit as per manufacturer’s instructions. cDNA was then synthesized using the GoScript^™^ Reverse Transcriptase kit (A5000, Promega) as per manufacturer’s instructions. qRT-PCR was performed to evaluate the relative differences in gene expression. The primers used are listed in Table S2. Results were analysed using the 2^−ΔΔCT^ method relative to the expression of RNA polymerase II.

### Western blot

Cells were seeded in 6-well plates and treated with TGFβ or TGFβ and AMC303 for 24 h. Cells were harvested and lysed in ice-cold RIPA buffer supplemented with PhosSTOP^™^ and a protease inhibitor cocktail (11836170001, Roche) by continuous agitation at 4 °C for 30 min. The cells were then centrifuged for 20 min at 12,000 rpm, and the pellet was discarded. The concentration of protein was then measured using Bradford reagent (500–0006, Biorad). A total of 5 µg of protein was loaded into a 10% SDS gel. The gel was run at 80 V for 10 min and then 200 V for 35 min. The protein from the gel was transferred onto a 0.2-µM nitrocellulose membrane (10600006, Amersham) at 60 V for 60 min. The membrane was blocked in 5% BSA for 2 h at room temperature and then incubated overnight at 4 °C in the primary antibody diluted in 3% BSA. A goat-anti-rabbit secondary antibody (G21234, Thermo Fisher Scientific, 1:2000) was used, and the target proteins were detected using a chemiluminescence detection kit (WBKLS0500, Millipore) according to the manufacturer’s instructions. Proteins were visualized using a chemiluminescence imaging system (Fusion Solo X, Vilber). Protein expression was quantified using ImageJ.

### Cotreatment of human cell lines and organoids

For cotreatment experiments, 200-nM AMC303 was applied to the wells. This was either as a single agent or in combination with TKIs/chemotherapy. Control wells were treated with DMSO (D2660, Sigma Aldrich). Cells were pretreated with 200-nM AMC303 for 3 days, with media and AMC303 replenishment every 24 h, followed by treatment with the TKI/chemotherapeutic agent for 4 days. At the end of 7 days, cells were fixed with 100% methanol for 10 min and then stained with Giemsa for 20 min. The Giemsa was then dissolved in 10% acetic acid, and the colour of the solution was measured using a spectrophotometer. The same treatment regimen was followed for cotreatment of cholangiocarcinoma organoids with AMC303 and gemcitabine. The cell viability was measured using the CytoTox-Glo^™^ Cytotoxicity Assay (G9290, Promega) as per the manufacturer’s instructions.

### Statistical analysis

All experiments were performed with at least three biological and three technical replicates. Statistical evaluation was performed by using GraphPad Prism version 9 (GraphPad Software, Inc., La Jolla, CA, USA). Student’s *t*-test with Welch’s correction was used to calculate the statistical significance of experiments. Data was presented at the mean ± standard deviation (SD) or standard error of the mean (SEM) as warranted. A *p*-value < 0.05 was considered statistically significant.

## Supplementary information

Below is the link to the electronic supplementary material.

ESM1 (DOCX 918 KB)

## Data Availability

The data supporting the results of this study are available on request from the corresponding author.
