# Supplementary material for: Peptide-based inhibition of CD44v6 renders liver carcinomas more susceptible to therapeutic intervention
Source: J Mol Med (Berl). 2025 Oct 31;103(11-12):1583–98. doi: 10.1007/s00109-025-02601-5 (PMC12675676; doi:10.1007/s00109-025-02601-5)
Supplement: Supplementary file 1 — (DOCX 918 KB) [file 109_2025_2601_MOESM1_ESM.docx]

**Supplementary File**

**Peptide-based inhibition of CD44v6 renders liver carcinomas more susceptible to therapeutic intervention**

Akshaya Srikanth, Ranjitha Vishnu Anand Rao, Rui Dong, Umesh Tharehalli, Thomas F.E. Barth, Klaus Dembowsky, Thomas Seufferlein, Reinhold Schirmbeck, André Lechel

**Table of contents:**

| Title | Description | Page number |
| --- | --- | --- |
| Fig. S1 | AMC303 does not have an effect on the tumorigenic potential of CD44v6^-^ cells | 2 |
| Fig. S2 | Hepatocyte Growth Factor (HGF) levels in liver cancer cell lines | 3 |
| Fig. S3 | Full length western blots (SNU423 lysates) | 4 |
| Fig. S4 | Full length western blots (SNU449 lysates) | 5 |
| Fig. S5 | Full length western blots (TFK1 lysates) | 5 |
| Fig. S6 | Full length western blots (SZ1 lysates) | 7 |
| Table S1 | Role of CD44v6 in different human tumour entities | 8 |
| Table S2 | Human primer pairs used for qRT-PCR | 9 |
| References | References for Table S1 | 10 |

**Fig. S1**


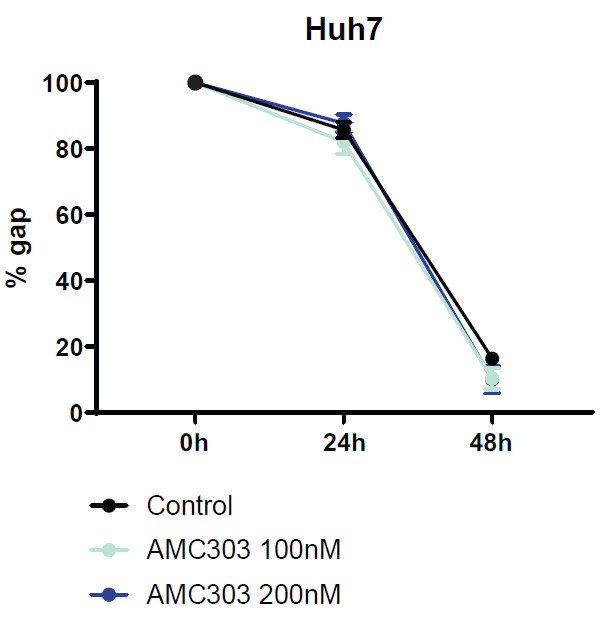


**Fig. S1: AMC303 does not have an effect on the tumorigenic potential of CD44v6-negative cells.** Migratory potential of Huh7, a CD44v6^-^ cell line, is unaffected upon treatment with AMC303. n=3 for all experiments.

**Fig. S2**

**Fig. S2:** HGF concentration in the conditioned medium of the liver cancer cell lines at 24h, 48h and 72h post seeding. n=2 for all experiments.

**Fig. S3**


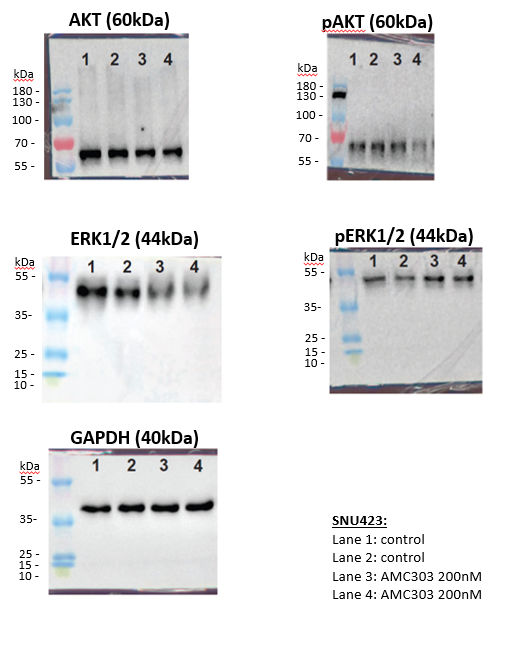


**Fig. S3:** Full length western blots (SNU423 lysates) for AKT, pAKT, ERK1/2, pERK1/2, and GAPDH. The full length western blots correspond to the cropped western blots shown in the main Figure 2F.

**Fig. S4**


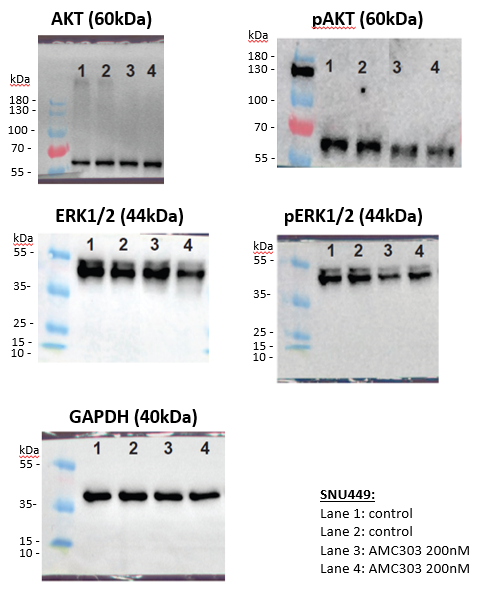


**Fig. S4:** Full length western blots (SNU449 lysates) for AKT, pAKT, ERK1/2, pERK1/2, and GAPDH. The full length western blots correspond to the cropped western blots shown in the main Figure 2F.

**Fig. S5**


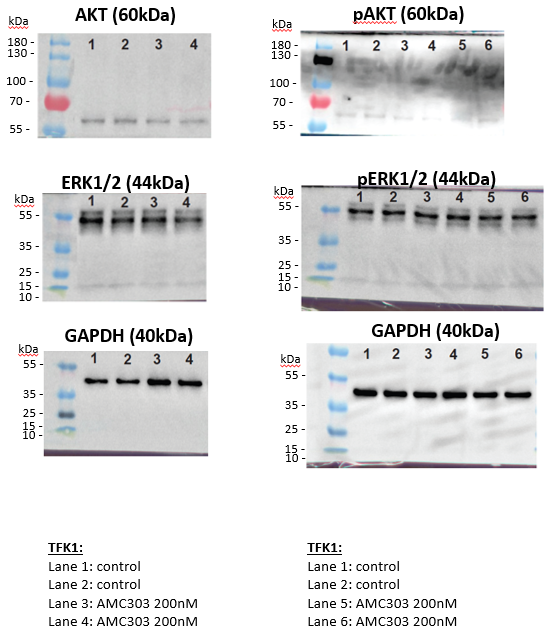


**Fig. S5:** Full length western blots (TFK1 lysates) for AKT, pAKT, ERK1/2, pERK1/2, and GAPDH. The full length western blots correspond to the cropped western blots shown in the main Figure 2F. Lane 3+4 from the WBs on the right side correspond to a different experiment and are not shown in the main figures.

**Fig. S6**


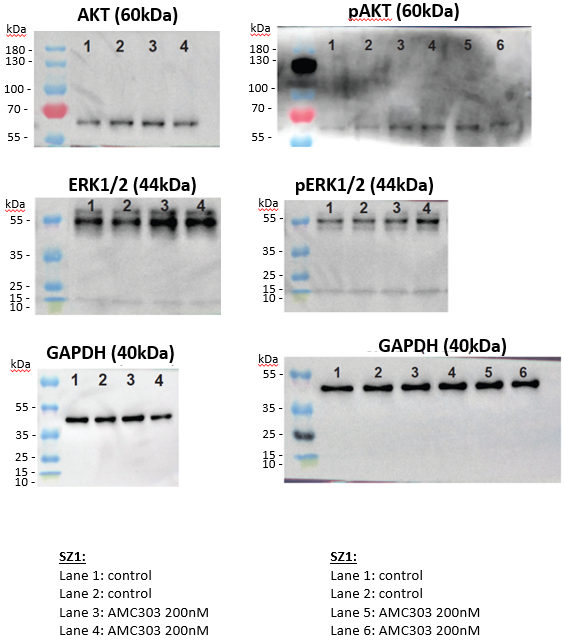


**Fig. S6:** Full length western blots (SZ1 lysates) for AKT, pAKT, ERK1/2, pERK1/2, and GAPDH. The full length western blots correspond to the cropped western blots shown in the main Figure 2F. Lane 3+4 from the WBs on the right side correspond to a different experiment and are not shown in the main figures.

**Table S1: Role of CD44v6 in different human tumour entities**

| Type of cancer | Outcomes/findings | References |
| --- | --- | --- |
| Breast cancer | CD44v6 expression is associated with poor prognosis. | [1-3] |
|  | CD44v6 expression is associated with increased tumour aggressiveness. | [2-9] |
|  | CD44v6 expression is not associated with tumour progression, aggressive disease and prognosis. | [10-14] |
| Colorectal cancer | CD44v6 expression confers poor overall survival. | [15-19] |
|  | CD44v6 is associated with increased drug resistance. | [20-24] |
|  | CD44v6 expression results in increase migration and invasion, and thereby development of more aggressive tumours. | [20, 25-28] |
|  | CD44v6 is not linked to poor outcomes. | [29, 30] |
| HNSCC | CD44v6 expression correlates with reduced overall and disease-free survival. | [31-36] |
|  | CD44v6 expression increases resistance to standard therapy. | [34, 37] |
|  | CD44v6 expression leads to increased migratory and invasive potential of tumours. | [38, 39] |
|  | CD44v6 is not related to tumour formation, progression and poor prognosis. | [40-42] |
| Liver cancer | CD44v6 expression is associated with poor patient prognosis. | [43, 44] |
|  | CD44v6 plays a role in malignant progression of liver cancer. | [43, 45-49] |
|  | Expression of CD44v6 is not a prognostic factor. | [48] |
| Pancreatic cancer | Increased levels of CD44v6 corresponds to shorter patient survival times. | [50-56] |
|  | CD44v6 expression is associated with increased tumour aggressiveness and metastasis. | [52, 54-65] |
|  | CD44v6 is a therapeutic target. | [56, 66] |

**Table S2: Human primer pairs used for qRT-PCR**

| **Gene** | **Sense Primer (5’-3’)** | **Antisense Primer (5’-3’)** |
| --- | --- | --- |
| E-Cadherin | TGCCCAGAAAATGAAAAAGG | GGATGACACAGCGTGAGAGA |
| NANOG | AGATGCCTCACACGGAGACT | AAGTGGGTTGTTTGCCTTTG |
| OCT3/4 | GAAGGATGTGGTCCGAGTGT | GCCTCAAAATCCTCTCGTTG |
| RNA Pol II | GTACATGCACTTGCCACAGACA | TCTCACTCAGCACCCGCATC |
| SNAI2 (Slug) | ACAGCGAACTGGACACACAT | CACAGTGATGGGGCTGTATG |
| Vimentin | GACAATGCGTCTCTGGCACGTCTT | TCCTCCGCCTCCTGCAGGTTCTT |
| ZEB1 | AAAGATGATGAATGCGAGTC | TCCATTTTCATCATGACCAC |

**References**

1. Yu, P., L. Zhou, W. Ke, and K. Li, *Clinical significance of pAKT and CD44v6 overexpression with breast cancer.* Journal of Cancer Research and Clinical Oncology, 2010. **136**(8): p. 1283-1292.

2. Wu, X.-J., X.-D. Li, H. Zhang, X. Zhang, Z.-H. Ning, Y.-M. Yin, and Y. Tian, *Clinical significance of CD44s, CD44v3 and CD44v6 in breast cancer.* Journal of International Medical Research, 2015. **43**(2): p. 173-179.

3. Qiao, G.-L., L.-N. Song, Z.-F. Deng, Y. Chen, and L.-J. Ma, *Prognostic value of CD44v6 expression in breast cancer: a meta-analysis.* OncoTargets and Therapy, 2018. **Volume 11**: p. 5451-5457.

4. Kopp, R., S. Classen, H. Wolf, P. Gholam, K. Possinger, and W. Wilmanns, *Predictive relevance of soluble CD44v6 serum levels for the responsiveness to second line hormone- or chemotherapy in patients with metastatic breast cancer.* Anticancer Research, 2001. **21(4B)**: p. 5.

5. Ma, W., Y. Dengy, and L. Zhouy, *The Prognostic Value of Adhesion Molecule CD44v6 in Women with Primary Breast Carcinoma: A Clinicopathologic Study.* Clinical Oncology, 2005. **17**: p. 5.

6. Afify, A., M.A. McNiel, J. Braggin, H. Bailey, and A.F. Paulino, *Expression of CD44s, CD44v6, and Hyaluronan Across the Spectrum of Normal-hyperplasia-carcinoma in Breast.* Appl Immunohistochem Mol Morphol, 2008. **2**: p. 7.

7. Bellerby, R., C. Smith, S. Kyme, J. Gee, U. Günthert, A. Green, E. Rakha, P. Barrett-Lee, and S. Hiscox, *Overexpression of Specific CD44 Isoforms Is Associated with Aggressive Cell Features in Acquired Endocrine Resistance.* Frontiers in Oncology, 2016. **6**.

8. Hu, S., M. Cao, Y. He, G. Zhang, Y. Liu, Y. Du, C. Yang, and F. Gao, *CD44v6 Targeted by miR-193b-5p in the Coding Region Modulates the Migration and Invasion of Breast Cancer Cells.* Journal of Cancer, 2020. **11**(1): p. 260-271.

9. Rustamadji, P., E. Wiyarta, and K.A. Bethania, *CD44 Variant Exon 6 Isoform Expression as a Potential Predictor of Lymph Node Metastasis in Invasive Breast Carcinoma of No Special Type.* Int J Breast Cancer, 2021.

10. Tempfer, C., A. Lösch, H. Heinzl, G. Häusler, E. Hanzal, H. Kölbl, G. Breitenecker, and C. Kainz, *Prognostic value of immunohistochemically detected CD44 isoforms CD44v5, CD44v6 and CD44v7–8 in human breast cancer.* European Journal of Cancer, 1996. **32**(11): p. 2.

11. Jansen, R.H., S.R. Joosten-Achjanie, J.W. Arends, A. Volovics, P.S. Hupperets, H.C. Schouten, and H.F. Hillen, *CD44v6 is not a prognostic factor in primary breast cancer.* Annals of Oncology, 1998. **9 (1)**: p. 2.

12. Tokue, Y., Y. Matsumura, N. Katsumata, T. Watanabe, D. Tarin, and T. Kakizoe, *CD44 Variant Isoform Expression and Breast Cancer Prognosis.* Japanese Journal of Cancer Research, 1998. **89**(3): p. 283-290.

13. Morris, S.F., D.M. O'Hanlon, R. McLaughlin, T. McHale, G.E. Connolly, and H.F. Given, *The prognostic significance of CD44s and CD44v6 expression in stage two breast carcinoma: an immunohistochemical study.* 2001. **27**: p. 4.

14. Berner, H.S., Z. Suo, B. Risberg, K. Villman, M.G. Karlsson, and J.M. Nesland, *Clinicopathological associations of CD44 mRNA and protein expression in primary breast carcinomas.* Histopathology, 2003. **42**(6).

15. Ropponen, K.M., M.J. Eskelinen, P.K. Lipponen, E. Alhava, and V.M. Kosma, *Expression of CD44 and variant proteins in human colorectal cancer and its relevance for prognosis.* Scand J Gastroenterol, 1998. **33**(3): p. 9.

16. Wielenga, V.J., R.v.d. Voort, J.W. Mulder, P.M. Kruyt, W.F. Weidema, J. Oosting, C.A. Seldenrijk, C.v. Krimpen, G.J. Offerhaus, and S.T. Pals, *CD44 splice variants as prognostic markers in colorectal cancer.* Scand J Gastroenterol, 1998. **33**(1): p. 5.

17. Vizoso, F.J., J.C. Fernandez, M.D. Corte, M. Bongera, R. Gava, M.T. Allende, J.L. Garcia-Muniz, and M. Garcia-Moran, *Expression and clinical significance of CD44V5 and CD44V6 in resectable colorectal cancer.* Journal of Cancer Research and Clinical Oncology, 2004. **130**(11): p. 679-686.

18. Wang, J.-L., W.-Y. Su, Y.-W. Lin, H. Xiong, Y.-X. Chen, J. Xu, and J.-Y. Fang, *CD44v6 overexpression related to metastasis and poor prognosis of colorectal cancer: A meta-analysis.* Oncotarget, 2017. **8**(8): p. 12866-12876.

19. Yan, B., Y. Mu, M. Cui, and L. Li, *Clinicopathological significance and prognostic implication of CD44 and its splice variants (v3 and v6) in colorectal cancer.* Translational Cancer Research, 2020. **9**(2): p. 9.

20. Bendardaf, R., H. Lamlum, R. Ristamäki, and S. Pyrhönen, *CD44 variant 6 expression predicts response to treatment in advanced colorectal cancer.* Oncology Reports, 2004. **11**(1): p. 4.

21. Ghatak, S., V.C. Hascall, N. Karamanos, R.R. Markwald, and S. Misra, *Chemotherapy induces feedback up-regulation of CD44v6 in colorectal cancer initiating cells through β-catenin/MDR1 signaling to sustain chemoresistance.* Frontiers in Oncology, 2022. **12**.

22. Lv, L., H.-G. Liu, S.-Y. Dong, F. Yang, Q.-X. Wang, G.-L. Guo, Y.-F. Pan, and X.-H. Zhang, *Upregulation of CD44v6 contributes to acquired chemoresistance via the modulation of autophagy in colon cancer SW480 cells.* Tumor Biology, 2016. **37**(7): p. 8811-8824.

23. Nicolazzo, C., F. Loreni, S. Caponnetto, V. Magri, A.R. Vestri, R. Zamarchi, A. Gradilone, A. Facchinetti, E. Rossi, E. Cortesi, and P. Gazzaniga, *Baseline CD44v6-positive circulating tumor cells to predict first-line treatment failure in patients with metastatic colorectal cancer.* Oncotarget, 2020. **11**(45): p. 4115-4122.

24. Wang, Q.-X., L. Lv, D.-R. Ye, Y.-H. Sun, X.-X. Pan, A. Bhandari, X.-H. Zhang, Ou-ChenWang, and H.-G. Liu, *Downregulation of CD44v6 Enhances Chemosensitivity by Promoting Apoptosis and Inhibiting Autophagy in Colorectal Cancer HT29 Cells.* Annals of Clinical & Laboratory Science, 2019. **49**(4).

25. Mulder, J.W., P.M. Kruyt, M. Sewnath, J. Oosting, C.A. Seldenrijk, W.F. Weidema, G.J. Offerhaus, and S.T. Pals, *Colorectal cancer prognosis and expression of exon-v6-containing CD44 proteins.* Lancet, 1994. **344**: p. 2.

26. Todaro, M., M. Gaggianesi, V. Catalano, A. Benfante, F. Iovino, M. Biffoni, T. Apuzzo, I. Sperduti, S. Volpe, G. Cocorullo, G. Gulotta, F. Dieli, Ruggero, and G. Stassi, *CD44v6 Is a Marker of Constitutive and Reprogrammed Cancer Stem Cells Driving Colon Cancer Metastasis.* Cell Stem Cell, 2014. **14**(3): p. 342-356.

27. Coppola, D., M. Hyacinthe, L. Fu, A.B. Cantor, R. Karl, J. Marcet, D.L. Cooper, S.V. Nicosia, and H.S. Cooper, *CD44V6 expression in human colorectal carcinoma.* Human Pathology, 1998. **29**(6): p. 8.

28. Gotley, D.C., J. Fawcett, M.D. Walsh, J.A. Reeder, D.L. Simmons, and T.M. Antalis, *Alternatively spliced variants of the cell adhesion molecule CD44 and tumour progression in colorectal cancer.* British Journal of Cancer, 1996. **74**(3): p. 9.

29. Koretz, K., P. Möller, T. Lehnert, U. Hinz, H.F. Otto, and C. Herfarth, *Effect of CD44v6 on survival in colorectal carcinoma.* Lancet, 1995. **345**(8945): p. 2.

30. Avoranta, S.T., E.A. Korkeila, K.J. Syrjänen, S.O. Pyrhönen, and J.T.T. Sundström, *Lack of CD44 variant 6 expression in rectal cancer invasive front associates with early recurrence.* World J Gastroenterol, 2012. **18**(33): p. 9.

31. Chai, L., H. Liu, Z. Zhang, F. Wang, Q. Wang, S. Zhou, and S. Wang, *CD44 Expression Is Predictive of Poor Prognosis in Pharyngolaryngeal Cancer: Systematic Review and Meta-Analysis.* The Tohoku Journal of Experimental Medicine, 2014. **232**(1): p. 9-19.

32. Kawano, T., Y. Nakamura, S. Yanoma, A. Kubota, M. Furukawa, Y. Miyagi, and M. Tsukuda, *Expression of E-cadherin, and CD44s and CD44v6 and its association with prognosis in head and neck cancer.* Auris Nasus Larynx, 2004. **31**(1): p. 6.

33. Okuyama, K., H. Fukushima, T. Naruse, S. Yanamoto, H. Tsuchihashi, and M. Umeda, *CD44 Variant 6 Expression and Tumor Budding in the Medullary Invasion Front of Mandibular Gingival Squamous Cell Carcinoma Are Predictive Factors for Cervical Lymph Node Metastasis.* Pathology &amp; Oncology Research, 2019. **25**(2): p. 603-609.

34. Patel, U., S. Kannan, S.U. Rane, N. Mittal, P. Gera, A. Patil, S. Manna, V. Shejwal, V. Noronha, A. Joshi, V.M. Patil, K. Prabhash, and M.B. Mahimkar, *Prognostic and predictive roles of cancer stem cell markers in head and neck squamous cell carcinoma patients receiving chemoradiotherapy with or without nimotuzumab.* British Journal of Cancer, 2022. **126**(10): p. 1439-1449.

35. Rodrigo, J.P., F. Dominguez, C. Alvarez, A. Herrero, and C. Suarez, *Expression of E-cadherin, CD44s, and CD44v6 in laryngeal and pharyngeal carcinomas.* American Journal of Otolaryngology, 2003. **24**(6).

36. Yang, Q., Y. Liu, Y. Huang, D. Huang, Y. Li, J. Wu, and M. Duan, *Expression of COX-2, CD44v6 and CD147 and Relationship with Invasion and Lymph Node Metastasis in Hypopharyngeal Squamous Cell Carcinoma.* PLoS ONE, 2013. **8**(9): p. e71048.

37. Sagawa, K., N. Uwa, T. Daimon, M. Sakagami, and T. Tsujimura, *Expression of CD44 variant isoforms, CD44v3 and CD44v6, are associated with prognosis in nasopharyngeal carcinoma.* The Journal of Laryngology & Otology, 2016. **130**(9): p. 843-849.

38. Banghua, L., K. Weijia, G. Shusheng, Y. Chengzhang, W. Guangping, and Z. Lixin, *Relationship between the expression of CD44v6 and development, progress, invasion and metastasis of laryngeal carcinoma.* Current Medical Science, 2005. **25**(3): p. 351-353.

39. Wang, S., N. Ma, W. Zhao, K. Midorikawa, S. Kawanishi, Y. Hiraku, S. Oikawa, Z. Zhang, G. Huang, and M. Murata, *Inflammation-Related DNA Damage and Cancer Stem Cell Markers in Nasopharyngeal Carcinoma.* Mediators Inflammation, 2016.

40. Mack, B. and O. Gires, *CD44s and CD44v6 Expression in Head and Neck Epithelia.* PLoS ONE, 2008. **3**(10): p. e3360.

41. Hal, N.L.v., G.A.v. Dongen, M.S.-v. Walsum, G.B. Snow, and R.H. Brakenhoff, *Characterization of CD44v6 isoforms in head-and-neck squamous-cell carcinoma.* International Journal of Cancer, 1999. **82**(6): p. 8.

42. Spafford, M.F., J. Koeppe, Z. Pan, P.G. Archer, A.D. Meyers, and W.A. Franklin, *Correlation of tumor markers p53, bcl-2, CD34, CD44H, CD44v6, and Ki-67 with survival and metastasis in laryngeal squamous cell carcinoma.* Arch Otolaryngol Head Neck Surg, 1996. **122**(6): p. 5.

43. Jha, R.K., Q. Ma, S. Chen, H. Sha, and S. Ding, *Relationship of fibronectin and CD44v6 expression with invasive growth and metastasis of liver cancer.* Cancer Investigations, 2009. **27**(3): p. 4.

44. Fu, Y., Y. Geng, N. Yang, N. Zhu, C.-Z. Wang, X.-C. Su, and H.-B. Zhang, *CD44v6 expression is associated with a poor prognosis in Chinese hepatocellular carcinoma patients: A meta-analysis.* Clin Res Hepatol Gastroenterol, 2015. **39**(6): p. 3.

45. Endo, K. and T. Terada, *Protein expression of CD44 (standard and variant isoforms) in hepatocellular carcinoma: relationships with tumor grade, clinicopathologic parameters, p53 expression, and patient survival.* Journal of Hepatology, 2000. **32**(1): p. 6.

46. Gao, C., *Osteopontin-dependent CD44v6 expression and cell adhesion in HepG2 cells.* Carcinogenesis, 2003. **24**(12): p. 1871-1878.

47. Kon, J., H. Ooe, H. Oshima, Y. Kikkawa, and T. Mitaka, *Expression of CD44 in rat hepatic progenitor cells.* Journal of Hepatology, 2006. **45**(1): p. 8.

48. Mima, K., H. Okabe, T. Ishimoto, H. Hayashi, S. Nakagawa, H. Kuroki, K. Miyake, H. Takamori, T. Beppu, and H. Baba, *The expression levels of CD44v6 are correlated with the invasiveness of hepatocellular carcinoma in vitro, but do not appear to be clinically significant.* Oncology Letters, 2012. **3**(5): p. 1047-1051.

49. Xiao, C.-Z., Y.-M. Dai, H.-Y. Yu, J.-J. Wang, and C.-R. Ni, *Relationship between expression of CD44v6 and nm23-H1 and tumor invasion and metastasis in hepatocellular carcinoma.* World J Gastroenterol, 1998. **4**(5): p. 2.

50. Castella, E.M., A. Ariza, I. Ojanguren, J.L. Mate, X. Roca, A. Fernandez-Vasalo, and J.J. Navas-Palacios, *Differential expression of CD44v6 in adenocarcinoma of the pancreas: an immunohistochemical study.* Virchows Archiv, 1996. **429-429**(4-5).

51. Gotoda, T., Y. Matsumura, H. Kondo, D. Saitoh, Y. Shimada, T. Kosuge, Y. Kanai, and T. Kakizoe, *Expression of CD44 Variants and Its Association with Survival in Pancreatic Cancer.* Japanese Journal of Cancer Research, 1998. **89**(10): p. 1033-1040.

52. Li, Z., K. Chen, P. Jiang, X. Zhang, X. Li, and Z. Li, *CD44v/CD44s expression patterns are associated with the survival of pancreatic carcinoma patients.* Diagnostic Pathology, 2014. **9**(1): p. 79.

53. Wang, H., S. Rana, N. Giese, M.W. Büchler, and M. Zöller, *Tspan8, CD44v6 and alpha6beta4 are biomarkers of migrating pancreatic cancer-initiating cells.* International Journal of Cancer, 2013. **133**(2): p. 10.

54. Zhou, G., D. Chiu, D. Qin, L. Niu, J. Cai, L. He, D. Tan, and K. Xu, *Expression of CD44v6 and integrin-β1 for the prognosis evaluation of pancreatic cancer patients after cryosurgery.* Diagnostic Pathology, 2013. **8**(1): p. 146.

55. Rall, C.J. and A.K. Rustgi, *CD44 isoform expression in primary and metastatic pancreatic adenocarcinoma.* Cancer Research, 1995. **55**(9): p. 4.

56. Matzke-Ogi, A., K. Jannasch, M. Shatirishvili, B. Fuchs, S. Chiblak, J. Morton, B. Tawk, T. Lindner, O. Sansom, F. Alves, A. Warth, C. Schwager, W. Mier, J. Kleeff, H. Ponta, A. Abdollahi, and V. Orian-Rousseau, *Inhibition of Tumor Growth and Metastasis in Pancreatic Cancer Models by Interference With CD44v6 Signaling.* Gastroenterology, 2016. **150**(2): p. 513-525.e10.

57. Chen, K., Z. Li, P. Jiang, X. Zhang, Y. Zhang, Y. Jiang, Y. He, and X. Li, *Co-expression of CD133, CD44v6 and human tissue factor is associated with metastasis and poor prognosis in pancreatic carcinoma.* Oncology Reports, 2014. **32**(2): p. 755-763.

58. Gaviraghi, M., P. Tunici, S. Valensin, M. Rossi, C. Giordano, L. Magnoni, M. Dandrea, L. Montagna, R. Ritelli, A. Scarpa, and A. Bakker, *Pancreatic cancer spheres are more than just aggregates of stem marker-positive cells.* Bioscience Reports, 2011. **31**(1): p. 45-55.

59. Jung, T., W. Gross, and M. Zöller, *CD44v6 Coordinates Tumor Matrix-triggered Motility and Apoptosis Resistance.* Journal of Biological Chemistry, 2011. **286**(18): p. 15862-15874.

60. Mu, W., Z. Wang, and M. Zöller, *Ping-Pong—Tumor and Host in Pancreatic Cancer Progression.* Frontiers in Oncology, 2019. **9**.

61. Pozza, E.D., I. Dando, G. Biondani, J. Brandi, C. Costanzo, E. Zoratti, M. Fassan, F. Boschi, D. Melisi, D. Cecconi, M.T. Scupoli, A. Scarpa, and M. Palmieri, *Pancreatic ductal adenocarcinoma cell lines display a plastic ability to bi-directionally convert into cancer stem cells.* International Journal of Oncology, 2015. **46**(3): p. 1099-1108.

62. Sun, H., S. Rana, Z. Wang, K. Zhao, M. Schnölzer, J. Provaznik, T. Hackert, Q. Lv, and M. Zöller, *The Pancreatic Cancer-Initiating Cell Marker CD44v6 Affects Transcription, Translation, and Signaling: Consequences for Exosome Composition and Delivery.* Journal of Oncology, 2019. **2019**: p. 1-24.

63. Wang, Z., A. Von Au, M. Schnölzer, T. Hackert, and M. Zöller, *CD44v6-competent tumor exosomes promote motility, invasion and cancer-initiating cell marker expression in pancreatic and colorectal cancer cells.* Oncotarget, 2016. **7**(34): p. 55409-55436.

64. Miyasaka, Y., E. Nagai, K. Ohuchida, K. Nakata, A. Hayashi, K. Mizumoto, M. Tsuneyoshi, and M. Tanaka, *CD44v6 expression in intraductal papillary mucinous neoplasms of the pancreas.* Pancreas, 2010. **39**(1): p. 4.

65. Heiler, S., Z. Wang, and M. Zöller, *Pancreatic cancer stem cell markers and exosomes - the incentive push.* World J Gastroenterol, 2016. **22**(26): p. 26.

66. Orian-Rousseau, V., *CD44, a therapeutic target for metastasising tumours.* European Journal of Cancer, 2010. **46**(7): p. 6.
